# Evolutionary Dynamics of the Ty3/Gypsy LTR Retrotransposons in the Genome of *Anopheles gambiae*

**DOI:** 10.1371/journal.pone.0016328

**Published:** 2011-01-24

**Authors:** Jose Manuel C. Tubio, Marta Tojo, Laia Bassaganyas, Georgia Escaramis, Igor V. Sharakhov, Maria V. Sharakhova, Cristian Tornador, Maria F. Unger, Horacio Naveira, Javier Costas, Nora J. Besansky

**Affiliations:** 1 Genes and Disease Programme, Center for Genomic Regulation, Barcelona, Spain; 2 Hospital Universitario de Santiago de Compostela, Santiago de Compostela, Spain; 3 Centro Nacional de Genotipado (CEGEN), Barcelona, Spain; 4 Department of Entomology, Virginia Tech, Blacksburg, Virginia, United States of America; 5 Eck Institute for Global Health, Department of Biological Sciences, University of Notre Dame, Notre Dame, Indiana, United States of America; 6 Departamento de Biología Celular y Molecular, Universidade da Coruña, A Coruña, Spain; 7 Fundación Pública Galega de Medicina Xenómica, Complexo Hospitalario Universitario de Santiago, Santiago de Compostela, Spain; Georgia Institute of Technology, United States of America

## Abstract

Ty3/gypsy elements represent one of the most abundant and diverse LTR-retrotransposon (LTRr) groups in the *Anopheles gambiae* genome, but their evolutionary dynamics have not been explored in detail. Here, we conduct an *in silico* analysis of the distribution and abundance of the full complement of 1045 copies in the updated AgamP3 assembly. Chromosomal distribution of Ty3/gypsy elements is inversely related to arm length, with densities being greatest on the X, and greater on the short versus long arms of both autosomes. Taking into account the different heterochromatic and euchromatic compartments of the genome, our data suggest that the relative abundance of Ty3/gypsy LTRrs along each chromosome arm is determined mainly by the different proportions of heterochromatin, particularly pericentric heterochromatin, relative to total arm length. Additionally, the breakpoint regions of chromosomal inversion 2La appears to be a haven for LTRrs. These elements are underrepresented more than 7-fold in euchromatin, where 33% of the Ty3/gypsy copies are associated with genes. The euchromatin on chromosome 3R shows a faster turnover rate of Ty3/gypsy elements, characterized by a deficit of proviral sequences and the lowest average sequence divergence of any autosomal region analyzed in this study. This probably reflects a principal role of purifying selection against insertion for the preservation of longer conserved syntenyc blocks with adaptive importance located in 3R. Although some Ty3/gypsy LTRrs show evidence of recent activity, an important fraction are inactive remnants of relatively ancient insertions apparently subject to genetic drift. Consistent with these computational predictions, an analysis of the occupancy rate of putatively older insertions in natural populations suggested that the degenerate copies have been fixed across the species range in this mosquito, and also are shared with the sibling species *Anopheles arabiensis*.

## Introduction

Transposable elements (TEs) are ubiquitous components of eukaryotic genomes, but differ widely in their abundance [1]. The vast majority of these mobile elements have deleterious effects on the host genome, because of the gene mutations and chromosomal rearrangements they promote, and usually they are efficiently eliminated by selection [2]. Purifying selection acts against TEs mainly in three ways: (1) against deleterious effects of TE insertions on neighboring genes [3], [4], (2) against deleterious effects of TE-generated expression products [5], and (3) against deleterious products of ectopic recombination among dispersed homologous TEs [6]. Nevertheless, each TE copy has a probability of fixation that depends not only on selective pressure, but also on host population size, and recombination frequencies in the surrounding host genomic DNA [7], [8].

LTR retrotransposons (LTRrs) are TEs characterized by the presence of long terminal direct repeats (LTRs) at their ends. These elements transpose by an intracellular “copy and paste” process involving an RNA intermediate, its reverse transcription, and integration of the resulting proviral DNA. After transposition, the new proviral copies of an LTRr family are generally full-length in size and presumably active, showing no divergence at the nucleotide level from the source sequence. However, over evolutionary time it is expected that these full-length copies diverge from the template, and become fragmented due to accumulation of indels [9] and solo-LTR formation [10], [11] by recombination between LTRs of the same retrotransposon.

Representative elements of the two main classes of TEs have been identified in the *An. gambiae* genome so far [12]. Recently, the comparative analysis of the genomes of the three most representative mosquitoes presented an update of their respective TE composition [13]: in the genome of *An. gambiae*, TEs of Class II represent 5.9% of the total genome size and Class I 6.85%, of which LTRrs constitutes about 2–3%. Within the LTRrs, Ty3/gypsy elements comprise the most abundant and diverse group, representing 1–1.5% of the total genome size, followed by Pao/Bel (0.98%) and Ty1/copia (0.15%). Previous studies [14], [15] reported that, relative to *Drosophila melanogaster* [16], the Ty3/gypsy group of *An. gambiae* is characterized by a larger proportion of degenerate copies, mainly heterochromatic. This suggested that an important fraction of Ty3/gypsy LTRr insertions have resided for a long time (as pseudogenes), leading to the prediction of high occupancy rates in the *An. gambiae* genome [14]. However, there was also evidence of recent activity by the majority of Ty3/gypsy element families in *An. gambiae*. The activity of LTRrs in a genome could have important functional consequences arising not only from the transposition itself, but also from the fact that LTRrs promote chromosomal rearrangements and may act as enhancers and promoters for gene transcription [3], [17]. These mechanisms could be responsible for the advent of novel functions and, in the case of *An. gambiae* (one of the most important vectors of human malaria), could alter genes that contribute to epidemiologically relevant aspects of mosquito physiology or behavior, making the analysis of the associations between LTRr insertions and genes especially interesting. Here, we have performed a detailed *in silico* analysis of the patterns of distribution, abundance and occupancy of Ty3/gypsy LTRrs across different structural and functional compartments of the updated *An. gambiae* genome assembly (AgamP3) [18], to yield insight into the evolutionary dynamics between these LTRrs and the *An. gambiae* genome.

## Results and Discussion

### Chromosomal distribution of Ty3/gypsy LTRrs

**Figure 1:**
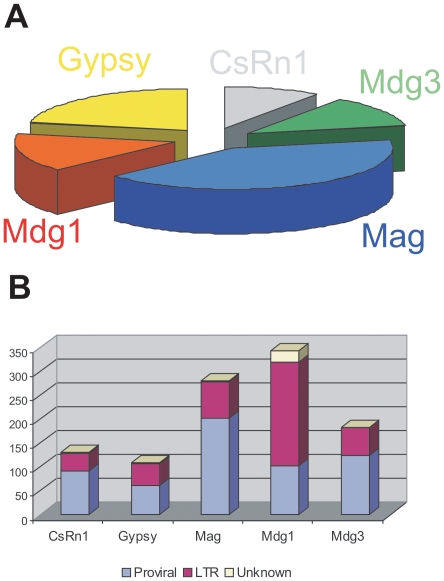
The Ty3/gypsy group of LTR retrotransposons in *An. gambiae*. The most abundant type of LTRrs in the genome of *An. gambiae* is the Ty3/gypsy group, also referred to as *Metaviridae* according to virus taxonomy [51]. So far, six different lineages of this group have been identified in insects based on the phylogenetic analysis of their reversetranscriptase, ribonuclease H, and integrase amino acid domains [52], [53], and designated CsRn1, Gypsy, Mag, Mdg1, Mdg3 and Osvaldo. (A) Our study in the PEST reference genome of *An. gambiae* confirmed the presence of all the insect lineages of the Ty3/gypsy group except Osvaldo, grouped in 73 well-represented families. The Mag linage is the most diverse, presenting 41% of the total set of families, followed by Mdg3 (22%), Mdg1 (15%), Gypsy (12%) and CsRn1 (10%). (B) We analyzed a total of 1045 insertions belonging to these 73 families in *An. gambiae*. The Mdg1 linage is the most abundant with 343 copies, most of them solo-LTRs. In the other four lineages the number of proviral copies exceeds the number of solo-LTRs. About 3% of the total amount of copies analyzed was not classified, mainly due to the presence of unsequenced gaps.

A total of 1045 insertions were identified belonging to 73 families of the Ty3/gypsy group of LTRrs in the genome of *An. gambiae* (Figure 1 and Dataset S1), including 166 copies undetected in previous analyses of this group of TEs [14], [15]. These insertions represent the bulk of the Ty3/gypsy complement in this genome, and constitute 1.17% of the total assembled genome size (3,197,007 bp out of 273,093,681 [19]). Each element was mapped *in silico* onto the AgamP3 assembly [18], an increase of 191 mapped insertions with respect to previous work [14], [15].

Excluding the two insertions detected on the unassembled Y chromosome (the Y chromosome is entirely heterochromatic and does not polytenize), the average density of Ty3/gypsy LTRrs per chromosome arm in terms of number of insertions per million bases (Mb) is 5.67 on the X and varies from 2.93 to 3.72 on the autosomes (Table 1). Taking into account that these insertions vary in size depending on their condition (i.e., fragmented or complete), the percentage of each arm composed of these LTRrs is 2.0 on the X and varies from 0.64 to 1.12 on the autosomes. Thus, the X chromosome contains 17% of all the mapped insertions, despite that it comprises only 10% of the total assembled chromosome complement. These estimates are consistent with a general overrepresentation of TEs on the X chromosome of *An. gambiae* (χ^2^ = 36.39, *P*≪0.01), as reported previously for the MOZ2 [12], [15] and AgamP3 [20] assemblies. In addition, autosomal density of these elements is inversely related to the size of each chromosome arm, being greater in the shorter arms 2L and 3L (χ^2^ = 5.08, *P* = 0.02).

**Table 1:** A comparative overview of Ty3/gypsy complement composition between chromosome arms of the AgamP3 assembly.

Arm region	LTRrs sequence (bp)	% of region	Number of insertions	Insertions per Mb	Insertions in clusters	Arm size (Mb)
2L	553,250	1.12	169	3.42	64	49.36
2R	554,625	0.90	187	3.04	48	61.55
3L	428,819	1.02	156	3.72	56	41.96
3R	342,671	0.64	156	2.93	24	53.20
X	487,729	2.00	138	5.67	54	24.40
Total	2,367,094	1.03	806	3.50	246	230.47
Total_UNK_	3,462	-	4	-	2	0.86

For each chromosome arm column 2 shows the total base pairs of Ty3/gypsy LTRrs and column 3 the % of each arm they represent. Columns 4 and 5 show the total number of insertions and the number of insertions per Mb of each region, respectively. Column 6 shows the number of insertions located 10 kb of each other. Finally, column 7 gives the total size of the chromosome region being analyzed. Last row (Total_UNK_) shows the information related to insertions located in euchromatin-heterochromatin intermediate regions that were not classified into the specific chromatin types showed in Tables 2 and 3.

**Table 2:** Characterization of the Ty3/gypsy complement of the AgamP3 heterochromatin.

Arm region	LTRrs sequence (bp)	% of region	Number of insertions	Insertions per Mb	Proviral versus Solo-LTR	% Proviral fragmented	Average divergence	Insertions in clusters	Genes per Mb	Region size (Mb)
2L_H_	280,640	8.93	70	22.28	50/19	72.0	2.54±2.65	39	13.05	3.14
2L_PH_	232,291	9.55	56	23.03	42/13	75.0	2.41±2.53	32	12.34	2.43
2L_DIH_	48,349	6.81	14	19.72	8/6	57.1	3.0±3.15	7	15.49	0.71
2R_H(PH)_	147,873	5.77	32	12.5	25/7	47.1	2.43±2.05	13	12.11	2.56
3L_H_	198,661	7.69	53	20.53	32/21	69.6	2.50±2.32	34	16.65	2.59
3L_PH_	145,321	8.01	36	19.83	22/14	80.0	2.46±2.25	26	18.18	1.82
3L_DIH_	53,340	6.95	17	22.16	10/7	50.0	2.58±2.52	8	13.04	0.77
3R_H_	108,438	2.77	50	12.79	20/30	75.0	2.40±2.68	14	14.83	3.91
3R_PH_	63,439	6.11	17	16.36	13/4	81.8	1.97±2.30	5	22.14	1.04
3R_CIH_	44,999	1.56	33	11.49	7/26	60.0	2.76±2.99	9	12.18	2.87
X_H(PH)_	357,360	8.15	85	19.39	54/29	63.6	2.46±2.98	35	12.78	4.38
Total_H_	1,092,972	6.59	290	17.49	182/106	66.2	2.47±2.62	135	13.81	16.58
Total_PH_	946,284	7.74	226	18.48	157/67	68.1	2.40±2.57	111	14.15	12.23
Total_DIH_	101,689	6.89	31	20.99	18/13	53.3	2.79±2.80	15	14.22	1.48
Total_CIH_	44,999	1.56	33	11.49	7/26	60.0	2.76±2.99	9	12.18	2.87

Different heterochromatic regions were analyzed in detail: heterochromatin (H), pericentric heterochromatin (PH), diffuse intercalary heterochromatin (DIH), compact intercalary heterochromatin (CIH). Column 2 shows the total base pairs of Ty3/gypsy LTRrs and column 3 the % of each arm they represent. Columns 4 and 5 show the total number of insertions and the number of insertions per Mb of each region, respectively. Column 6 gives the number of insertions of each condition category (proviral and solo-LTR), and column 7 the percentage of proviral copies that are fragmented. Column 8, the average divergence (from the consensus of the corresponding family) of the Ty3/gypsy LTRrs contained in each chromosome region, and the standard deviation. Column 9, the number of insertions located within 10 Kb of each other. Column 10 gives gene density. Column 11 gives the total size of the chromosome region being analyzed.

**Table 3:** Characterization of the Ty3/gypsy complement of the AgamP3 euchromatin.

Arm region	LTRrs sequence (bp)	% of region	Number of insertions	Insertions per Mb	Proviral versus Solo-LTR	% Proviral fragmented	Average divergence	Insertions in clusters	Genes per Mb	Region size (Mb)
2L_E_	270,776	0.59	97	2.11	56/40	30.6	2.12±2.79	25	65.26	45.90
2L_PE_	49,198	1.64	14	4.66	9/5	50.0	2.25±2.86	0	69.00	3.00
2L_NPE_	221,578	0.52	83	1.93	47/35	26.2	2.09±2.81	25	65.01	42.90
2R_E_	406,752	0.69	155	2.63	92/60	42.1	2.07±2.74	35	64.00	58.97
2R_PE_	90,482	3.02	28	9.33	19/8	66.7	3.04±3.58	19	73.33	3.00
2R_NPE_	316,270	0.57	127	2.27	73/52	36.9	1.91±2.58	16	63.50	55.97
3L_E_	229,275	0.59	102	2.60	47/48	38.1	2.08±2.62	22	53.18	39.17
3L_PE_	7,175	0.24	10	3.33	4/4	33.3	1.79±1.79	3	57.33	3.00
3L_NPE_	222,100	0.61	92	2.54	43/44	38.5	2.10±2.70	19	52.84	36.17
3R_E_	233,438	0.48	105	2.14	45/58	30.0	1.77±2.57	10	50.69	40.06
3R_PE_	34,463	1.15	23	7.66	7/16	57.1	2.42±2.48	4	34.00	3.00
3R_NPE_	199,025	0.43	82	1.78	38/42	24.2	1.62±2.59	6	51.78	46.06
X_E_	130,369	0.65	53	2.66	23/30	43.8	1.72±2.26	19	51.89	19.93
X_PE_	53,285	1.78	15	5.00	8/7	57.1	1.29±1.75	8	48.00	3.00
X_NPE_	77,084	0.46	38	2.24	15/23	33.3	1.94±2.49	11	52.57	16.93
Total_E_	1,270,660	0.60	512	2.40	263/236	36.8	1.99±2.65	111	58.09	213.03
Total_PE_	234,603	1.56	90	6.00	47/40	56.8	2.27±2.71	34	56.33	15.00
Total_NPE_	1,036,057	0.52	422	2.13	216/196	32.4	1.94±2.64	77	58.22	198.03

Different euchromatic regions were analyzed in detail: euchromatin (E), pericentromeric euchromatin (PE) and non-pericentromeric euchromatin (NPE).

A total of 246 Ty3/gypsy insertions (30% of the total mapped) were clustered in the genome, where clustered is defined by insertions located within 10 Kb of each other (Table 1). The pericentromeric region of the polytene complement, where recombination rate is lower, harbors 68% of the total copies involved in clusters. Similar findings were reported in *Drosophila* [21], [22], indicating that low-recombining regions of the genome are prone to accumulation of TEs.

### Distribution and abundance of Ty3/gypsy LTRrs in the heterochromatin

**Figure 2:**
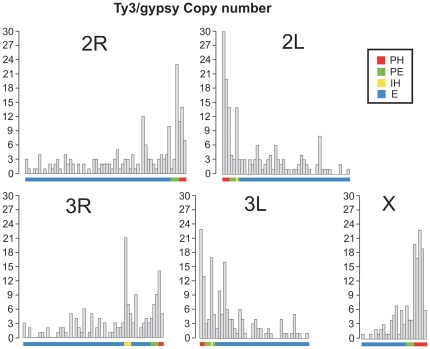
Chromosomal distribution of the Ty3/gypsy complement in the AgamP3 assembly. The distribution and relative abundance of the Ty3/gypsy insertions along chromosome arms are displayed in windows of 1 Mb. For each chromosome arm the distance from the centromere to the telomere is represented along the X axis. A coloured line under the X axis represents the different regions along each chromosome [23]: red indicates the PH; green the PE; yellow the IH; and the rest of the euchromatin is represented by a blue line. The number of insertions is represented along the left Y axis, from 0 to 30. Bars represent the abundance per Mb. See Tables 2 and 3 for details.

In terms of base pairs, the heterochromatin harbors 46% of the total mapped Ty3/gypsy complement in the genome, despite the fact that heterochromatin comprises only 7.2% of the total genome size [23]. The abundance and distribution of Ty3/gypsy LTRrs was characterized relative to the three main types of heterochromatin: pericentric (PH), diffuse intercalary (DIH), and compact intercalary (CIH) [23] (Table 2, Figure 2). The PH, which represents about three quarters of total heterochromatin size, contains 87% of the total bases of the Ty3/gypsy complement. The comparison between PH and intercalary heterochromatin (IH) shows that PH contains a higher density of Ty3/gypsy insertions (18.48 versus 14.72 insertions per Mb). This principal accumulation of insertions in the PH may result from lower rates of recombination adjacent to centromeres [24], [25].

The comparison between DIH and CIH also revealed different patterns (Table 2). First, Ty3/gypsy elements comprise 6.9% of the DIH size, but only 1.6% of the CIH. Interestingly, in the latter compartment solo-LTRs are 3.7 times more abundant than proviral insertions, a significant excess relative to any other region in the genome (euchromatic or heterochromatic). Although poorly defined at the molecular level, some peculiarities of CIH have been described. For instance, both the nuclear envelope protein lamin Dm_0_ and the non-histone chromosomal protein HP1 bind pericentric hetereochromatin, DIH, and some euchromatic regions, but not CIH [23]. Taking into account the scarce knowledge of the characteristics of CIH at the molecular level, the high proportion of solo-LTRs is difficult to interpret.

Overall, these data suggest that the relative abundance of Ty3/gypsy LTRrs along each chromosome arm of *An. gambiae* is determined mainly by the different proportions of heterochromatin, particularly PH, relative to total arm length. This is especially manifest on the X chromosome where PH—accounting for 18% of the total length—contains >60% of the Ty3/gypsy insertions (Table 2).

### Distribution and abundance of Ty3/gypsy LTRrs in the euchromatin

Within the euchromatin, gene density and recombination rate are expected to play an important role in the relative abundance and distribution of the Ty3/gypsy LTRrs [24], [26]. Both parameters vary along each euchromatic arm, being lower adjacent to the PH [20], [27], [28]. Ty3/gypsy complement represents 0.6% of the total mapped euchromatin of the AgamP3 assembly (Table 3, Figure 2). As expected, the number of Ty3/gypsy insertions is greater in the pericentric euchromatin (PE) of each arm (χ^2^ = 87.13, *P*≪0.01). The PE (defined as a 3 Mb region of euchromatin proximal to the PH on each arm) represents only 7% of the total size of the euchromatic complement but it contains 17% of the total number of Ty3/gypsy euchromatic insertions. Moreover, a hotspot for Ty3/gypsy LTRrs is found in the PE of 2R, where 18 insertions were clustered within a region of about 100 Kb (positions 58,001,186–58,104,526). Similar results were found for the PE of *D. melanogaster* [21], [22], where the average density of LTRrs is ∼4-fold higher relative to the overall average in euchromatin.

As was the case for heterochromatin, the euchromatic portion of the X chromosome also contains an overrepresentation of Ty3/gypsy insertions (χ^2^ = 8.635, *P* = 0.003). Gene density does not appear to explain this significant deviation (Table 3), thus it may be due to relaxed selective pressure against LTRrs in light of reduced opportunity for deleterious ectopic recombination on the X relative to autosomes in the population [2], [29].

Contrary to expectation, the non-pericentromeric euchromatin (NPE) of 3R presents the lowest density of Ty3/gypsy insertions despite having the lowest gene density (Table 3). These elements represent only 0.37% of the total NPE sequence on 3R. This, together with the fact that proviral insertions in 3R present the lowest degree of divergence among all chromosome arms (Dataset S1 and Table S1), is indicative of a faster turnover rate of proviral insertions. Interestingly, recent comparative physical mapping of congeners *An. funestus* and *An. stephensi* relative to *An. gambiae* (Sharakhova et al., unpublished data) revealed that only on 3R of *An. gambiae* are all identified syntenic blocks conserved among all three species, whereas the other autosome arms have syntenic blocks conserved only between species pairs. Lower gene density coupled with conserved microsynteny is a characteristic of genomic regulatory blocks [30]. These blocks extend over long distances and are constituted by central arrays of highly conserved non-coding elements of regulatory potential and their developmental regulated target genes, whose maintenance is fostered by selection against the accumulation of potentially deleterious TEs [31]. In *Drosophila*, the percentage of conserved genomic sequences laying in coding regions is lower than in non-coding ones [32]. Therefore, the putative presence of genomic regulatory blocks at 3R may explain the apparent contradiction of lowest gene density coupled with lowest Ty3/gypsy insertions.

### The role of inversions on the abundance and distribution of Ty3/gypsy LTRrs

The ectopic recombination model [6] suggests that the low recombination rates associated with regions adjacent to breakpoints in inversion heterozygotes [33] may reduce their deleterious effect on the host genome in those regions. Some empirical support for this model has been reported in insects, such as the *2j* inversion of *D. buzzatii* [22]. Specification of the precise location of the chromosomal breakpoints for inversion 2La [34] offers an opportunity to test the potential role of inversions as shelters for TEs in *An. gambiae*. It could be predicted that the entire rearrangement (not merely the breakpoint regions) may shelter TEs, because recombination is strongly reduced within the rearranged region in 2La/+a heterokaryotypes relative to 2La homokaryotypes, and somewhat reduced flanking the rearrangement as well [28]. The density of TEs along the 2L euchromatin (Dataset S2) was 3.08% within the limits of inversion 2La (coordinates 20,524,058–42,165,532), 5.32% between the proximal breakpoint and the pericentromeric region (coordinates 6,460,610–20,524,057), and 1.10% beyond the distal inversion breakpoint (coordinates 42,165,533–49,364,325). Although these densities differ significantly (*P*≪0.01), they counter the expectation that TEs have accumulated disproportionately within the limits of the inversion. However, both 50 kb regions immediately surrounding each breakpoint contained a highly significant (*P*≪0.01) increase in TE density relative to the rest of the euchromatin (13.21% and 8.53% for proximal and distal breakpoints). In addition, a 1 Mb region centered on the proximal breakpoint contained a significant increase in the number of Ty3/gypsy insertions relative to the rest of the NPE on this arm (χ^2^ = 13.57, *P*<0.01). Similarly, the density of Ty3/gypsy LTRrs is 3.3-fold higher in this 1 Mb region than the average for 2L euchromatin as a whole.

The overall data suggest that while the 2La rearrangement does not influence the overall abundance of TEs in the euchromatin of 2L, its breakpoints may do so. The role of inversions on TE distribution on chromosome 2R, where at least five rearrangements are common in complex configurations [35], remains to be investigated in detail.

### Structural variation (SV) and sequence divergence within Ty3/gypsy families

After transposition, the new copies of an LTRr family are generally full-length in size, and identical to the source sequence. The LTRrs of *An. gambiae* are typically 5 to 8 kb in length [15], and during their evolution it is expected that these full-length copies will become diverged in sequence and structure, due to solo-LTR formation and/or indel accumulation [16], [21], [26], [36].

**Figure 3:**
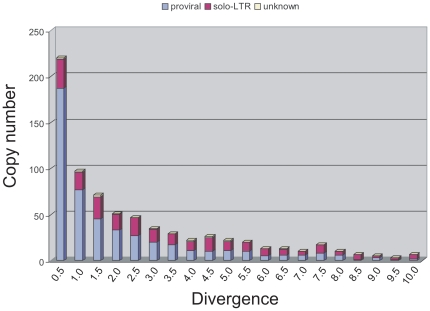
Intra-family divergence of Ty3/gypsy LTRrs in the genome of *An. gambiae*. As a general rule, those copies of LTRrs showing low sequence divergence from the consensus may be viewed as relatively recent transposition events in the genome, while high divergent copies may be considered remnants of older insertion events. This graph shows the relative abundance of Ty3/gypsy copies in the PEST genome relative to the divergence they present with respect to the corresponding consensus. Divergence is represented in a range from 0 to 10% in windows of 0.05. Proviral insertions are indicated in blue, solo-LTRs in red, and those unclassified (unknown) in yellow. The comparison between the group of insertions <2% divergent from the consensus, and those showing >5% divergence, reveals a significant decrease of the number of proviral copies versus solo-LTRrs (χ^2^ = 39.00, *P*≪0.001), suggesting that selection is more efficient acting against proviral copies rather than solo-LTRs, and/or it is more permissive with solo-LTRs allowing their persistence in the genome over time.

Assuming that consensus sequences represent full-length and active copies of the LTRr families [21], the level of divergence estimated between each copy and its family consensus provides an estimate of the age of insertion. Divergence could be estimated for 489 proviral LTRrs, 239 solo-LTRs, and 10 unclassified LTRrs (Dataset S1); the remaining 307 copies were excluded from the divergence analysis due to ambiguous alignments. The distribution of divergence values (Figure 3) indicates that 20% of solo-LTRs are ≥99% identical with their respective consensus, suggesting that solo-LTR formation [36] is an important mechanism for the inactivation of LTRr proviral insertions in their preliminary stages of evolution in the genome of this mosquito, as is the case for Ty1-like elements of the yeast *Saccharomyces cerevisiae* [10], [11]. In addition, 54% of the *An. gambiae* Ty3/gypsy proviral insertions are ≥99% identical with the consensus, suggesting that transposition events have occurred recently in about three-quarters of the Ty3/gypsy families [15]. On the other hand, 28% of the solitary-LTRs and 11% of the proviral insertions are diverged ≥5% from the consensus, indicating that an important fraction of the total number of LTRrs persist in the genome as pseudogenes from more ancient retroposition events.

Interestingly, the ratio of proviral to solo-LTR copies decreases with divergence (i.e., over time) (Figure 3). This suggests that selection is more efficient acting against proviral copies and/or is more permissive with solo-LTRs, given that proviral insertions are ∼17-fold larger than solo-LTRs [15] and may be more prone to ectopic recombination [26]. Consistent with this hypothesis, Tables 2 and 3 show a significant overall preponderance of proviral insertions versus solo-LTRs in the PH relative to the NPE (χ^2^ = 18.66, *P*≪0.001), presumably because recombination is reduced and selection against proviral copies is relaxed in the PH. Moreover, there is an overall higher divergence of the insertions located in the heterochromatin versus NPE (t = 5.91, *P* = 0.0004), implying a lower turnover rate and retention of older insertions in heterochromatin, allowing genetic drift to increase the occupancy of heterochromatic insertions in populations [2], [24], [25].

Structural variants were classified as “partial” whenever the sum of deletion lengths exceeded 3% of the consensus sequence. A total of 905 Ty3/gypsy insertions could be classified according to this criterion (140 copies were excluded due to unreliable alignments). Approximately two-thirds of these were partial elements. Among the set of partial elements, most (73%) were solo-LTRs and the remainder were partial derivatives of proviral copies. These proviral-partial copies contribute substantially to an increase in the average divergence of the Ty3/gypsy complement (Tables 2 and 3).

**Figure 4:**
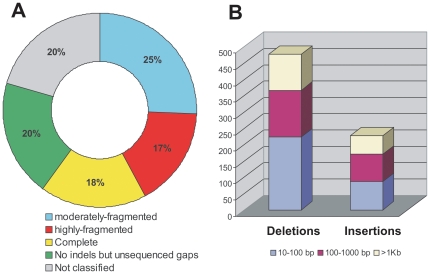
Patterns of SV of the Ty3/gypsy retrotransposons of *An. gambiae*. (A) We were able to classify 80% (462 out of 581) of the total number of proviral insertions of the Ty3/gypsy group according to their level of fragmentation (Dataset S1). The remaining 119 (20%) insertions were not classified due to unreliable alignments with respect to the respective consensus. We determined that 42% of the proviral insertions (246 out of 581) present any indel ≥10 bp: 148 (25%) are moderately-fragmented and 98 (17%) correspond to highly-fragmented insertions. On the other hand, 38% (216 out of 581) of the total number of proviral elements did not present any internal deletion or insertion ≥10 bp: 102 (18%) corresponded to true complete insertions, and the remaining 114 (20%) contain tracks of unsequenced gaps. Therefore, the total number of proviral copies with any level of fragmentation is probably underestimated. (B) We detected a total of 705 indels among the fragmented insertions of the Ty3/gypsy group of *An. gambiae*: 477 deletions and 228 insertions. Among deletions the most common are those with size in the range of 10–100 bp (47%), followed by those with size of 100 bp to 1 Kb (30%) and, finally, those longer than 1 Kb (23%). With respect to the insertions, the proportions were 38%, 37% and 25%, respectively.

More in-depth analysis of structural degradation was possible for 462 (80%) of the total proviral copies (Figure 4), of which roughly half (246) bear one or more indels ≥10 bp, and relatively few (102; 18%) are complete (i.e., without indels or unsequenced gaps). In general, the total number of deletions in an LTRr copy exceeds the number of insertions, indicating that deletions occur more frequently than insertions (477 versus 228), as reported for *D. melanogaster* [21], [26]. Accordingly, among the recent proviral copies (i.e., those ≥99% identical to the consensus), deletions are more frequent than insertions (63% versus 37%), being an important process for the inactivation of the proviral copies in the preliminary stages of their evolution in the genome, together with solo-LTR formation [10], [11].

### Ty3/gypsy LTR retrotransposon-gene associations

The distribution of TEs along the euchromatic arms should be the outcome of both new transposition events and the strength of natural selection acting differentially against these new mutations [37] according to their location (i.e., intergenic regions, exons or introns). In the NPE of *An. gambiae*, 33% (141) of the Ty3/gypsy insertions are associated with genes (Table 4 and Table S2), similar to the observed proportion in the *D. melanogaster* genome [37]. Most genes involved in these associations are annotated as novel protein-coding and only 9% are known protein-coding genes.

**Table 4:** Ty3/gypsy LTRr-gene associations in known protein coding genes in the PEST strain genome.

LTRr location	Div	Condition	Proximity in bp	Gene Name	Region	Putative Gene Function
2L: 13196772–13201826	0.0	Proviral	159	MYD	5′	TOLL pathway signalling
2L: 24637421–24637824	0.7	Solo-LTR	348	CPR29	5′	cuticular protein
2L: 37133058–37136178	6.2	Proviral	0	GPRGR28	Exon	Gustatory receptor
2L: 45978291–45978633	4.5	Proviral	306	LYSC3	3′	C-type lysozyme
2L: 24637421–24637824	0.7	Solo-LTR	389	CPR28	3′	cuticular protein
2R: 4043596–4047772	0.0	Proviral	0	GPRCCK2	Intron	Putative gastrin receptor
2R: 708350–713163	0.1	Proviral	600	GPRCAL2	5′	Putative calcitonin receptor
2R: 9916959–9920921	1.0	Proviral	0	GPRALS2	Intron	Putative allatostatin receptor
2R: 9987441–9991201	0.9	Proviral	0	GPRALS2	Intron	Putative allatostatin receptor
2R: 27194388–27194533	0.0	Solo-LTR	0	CLIPD8	Intron	Clip-domain serine protease
2R: 45978291–45978633	4.5	Proviral	410	LYSC8	5′	C-type lysozyme
2R: 54126431–54126786	0.0	Solo-LTR	620	MOC2A_ANOGA	5′	Molybdenum cofactor synthesis
2R: 54126431–54126786	0.0	Solo-LTR	957	MOC2B_ANOGA	5′	Molybdenum cofactor synthesis
3L: 33267375–33267630	3.2	Solo-LTR	759	CLIPA5	3′	Clip-domain serine protease
3R: 7766729–7766729	n. d.	Solo-LTR	404	TOLL1B	5′	Toll-like Receptor
X: 6253402–6258354	0.0	Proviral	0	GPRNPY	Intron	Putative neuropeptide Y receptor
X: 7234852–1191916	0.0	Proviral	118	TPX1	5′	Thioredoxin peroxidase

We identified 17 known protein genes associated with Ty3/gypsy insertions. Column 1 gives the coordinates in the Agam P3 assembly of each insertion associated with a gene; column 2 their divergence (%) from the consensus (n. d. means “not determined”); column 3 the condition category of each insertion; and column 4 the proximity to a gene in base pairs (a proximity of 0 bp means that the insertion is located within a gene). Column 5 shows the name of the gene involved in the association. Column 6 indicates the region of the gene affected: (i) exon or intron, if the insertion is located within the transcription boundaries; and (ii) 5′ and 3′, if the insertion is located downstream and upstream, respectively. Column 7 shows the putative function of the gene involved in the association.

Within gene boundaries, there was a significant excess of Ty3/gypsy copies located in introns. Three insertions were located in exons (Table 4), with potential functional implications. In addition, 17% of gene-associated insertions occur in the 1 kb region upstream or downstream of a gene, with potential regulatory implications [37].

For all gene-associated insertions located in the NPE, whose sequence divergence from the consensus could be estimated (114 of 141), 61% are putatively very recent (identity ≥99%), suggesting that they may be polymorphic in *An. gambiae* and vary geographically among populations [38]. Most of these putatively recent insertions (83%) are proviral, consistent with the overall pattern observed in the genome (Table S1).

### Fixation of TEs in the genome

As a preliminary test of conclusions drawn from the computational analysis of Ty3/gypsy in the PEST reference genome [14], [15], the level of heterozygosity (occupancy rate at an insertion site) was studied by PCR and sequencing in natural populations of *An. gambiae*. It was predicted that insertions in PEST suspected to be relatively ancient based on high sequence divergence from the family consensus would be fixed within and among natural populations. Eight Ty3/gypsy LTRr insertion sites were initially screened with small sample sizes, and three were subsequently screened in more depth (Table S3). The initial screening was conducted by PCR, employing twenty *An. gambiae* mosquitoes (five belonged to the M and five to the S molecular forms from West Africa, and ten belonged to the S form from East Africa). This low-resolution analysis allowed us to detect at least four insertions present in all the mosquitoes sampled. Among these four, three insertions (loci *GQ468821*, *GQ468822* and *GQ468823*) were inferred to be relatively old based on their high divergence to the consensus sequence (>7% for each of the three loci). Under this hypothesis, they were subjected to more extensive population analysis with the expectation of finding high occupancy rates throughout. By comparison, the occupancy rates of four additional insertions bearing very high sequence identity with the consensus suggested that they might prove to be population-specific in larger surveys.

**Figure 5:**
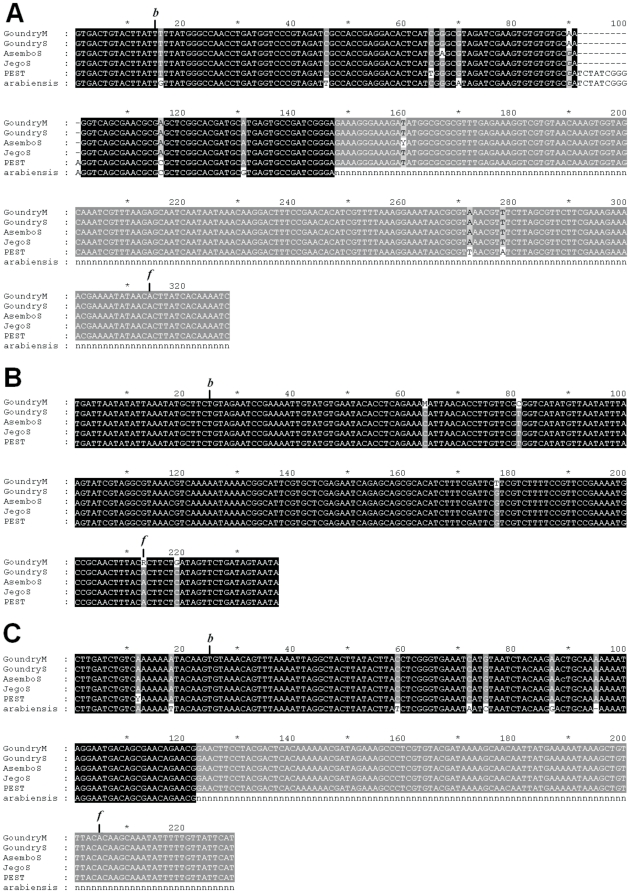
Fixation of Ty3/gypsy insertions in *Anopheles gambiae*. The extensive population and sequencing analyses of three highly divergent Ty3/gypsy insertions in *An. gambiae* confirmed their fixation in the species. The Gene Bank accession numbers for these loci are *GQ468823*, *GQ468822*, and *GQ468821*. Additionally, the presence of loci *GQ468823* and *GQ468821* were also confirmed in the closely related species *An. arabiensis*. This figure shows the multiple sequence alignments along the entire insertion and junctions obtained through an extensive population analysis. The DNA sequenced corresponds to mosquitoes of molecular forms M and S of *An. gambiae* from Goundry (Burkina Faso), two populations of the molecular form S from the villages of Asembo and Jego in Kenya (AsemboS and JegoS), and two populations of *An. arabiensis* from Burkina Faso and Kenya. In addition, the corresponding sequences from the PEST strain [12] are also shown. (A) Alignment of nucleotide sequences for locus *GQ468823.* Sequence of the LTRr copy extends from position 15 to 314. Target site duplication (CTTAT) corresponds to positions 9–14 and 315–319. Sequences of the junctions correspond to positions 1–8 and 320–328. “b” and “f” indicate the first and last positions of the insertion, respectively. (B) Alignment of nucleotide sequences for locus *GQ468822.* Sequence of the LTRr copy extends from position 25 to 213. Target site duplication (CTTC) corresponds to positions 21–24 and 214–217. Sequences of the junctions correspond to positions 1–20 and 218–237. (C) Alignment of nucleotide sequences for locus *GQ468821.* Sequence of the LTRr copy extends from position 25 to 205. Target site duplication (CAAG) corresponds to positions 21–24 and 206–209. Sequences of the junctions correspond to positions 1–20 and 210–229.

The presence of the three high-divergent Ty3/gypsy insertions was tested in 163 *An. gambiae* mosquitoes belonging to M and S populations from West Africa, and S populations from East Africa. In agreement with our expectations, genotyping surveys confirmed that these three insertions are present in all population samples of *An. gambiae*, showing an occupancy rate of 100%. Furthermore, we verified this observation in all the populations by sequencing of the amplicon, confirming the presence of the LTRr insertions in all the mosquitoes analyzed. The sequences of the junctions were >99% identical to the PEST reference [12] (Figure 5).

The long-term persistence of TEs in eukayotic genomes has been explained by models of selfish DNA evolution [2]. Briefly, on the one hand natural selection is expected to put limits to the total number of elements because of their deleterious effects. On the other hand, each specific retrotransposon insertion has a low probability of fixation, which depends on selective pressure, population size, and recombination frequencies in the surrounding genome. The three fixed insertions studied showed high sequence divergence relative to the consensus, indicative of an ancient origin. Loci *GQ468821* and *GQ468823* are located in intergenic regions of the NPE of 3R and 2L, respectively. Locus *GQ468822* was not mapped onto chromosomes, being probably heterochromatic according to the composition of the region [18]. The data available suggest that these insertions had a near-neutral evolution until became fixed by genetic drift. The overall preponderance of divergent fragmented insertions in *An. gambiae* relative to *D. melanogaster* has been interpreted as consequence of a major role of genetic drift in the mosquito genome [14].

### An. gambiae and An. arabiensis share TE-loci

LTRr insertions at loci *GQ468821* and *GQ468823* are also present in *An. arabiensis* as evidenced by genotyping and confirmed by sequencing (Figures 5A and 5C) in samples from West and East Africa (10 each). With respect to the LTR insertion at locus *GQ468821*, all the *An. arabiensis* specimens analyzed showed the expected amplicon size, revealing an occupancy rate of 100% (20/20). Unfortunately, the amplification of the LTRr insertion at locus *GQ468823* in *An. arabiensis* was only successful when using primers F1 and R2, and only in a few samples. Nevertheless, successful amplification of *locus GQ468823* in some *An. arabiensis* samples allowed us to obtain the sequence of the junctions and, therefore, to certify presence of the LTRr insertion in the genome of *An. arabiensis* (Figure 5A). Thus, these results suggest that the Ty3/gypsy-like insertions common to *An. gambiae* and *An. arabiensis* transposed into the genome before the split of both species or, alternatively, the insertions could be shared through recent genetic introgression [39]. Unfortunately, the limited data available, together with the fact that these two species have recently speciated and gene flow between them still persists, make difficult to distinguish unambiguously between introgression and the probable condition of ancestral insertions [40].

## Methods

### *In silico* identification of Ty3/gypsy insertions

The identification and family assignments of Ty3/gypsy LTRr copies were obtained following the criteria defined by Tubio et al. [15] with some refinements. Briefly, the canonical sequences of each family (or consensus sequences, when possible) were recruited from Repbase [41]. The canonical sequences represent complete copies putatively active, and the consensus sequences correspond to those constructed after alignment of at least three complete copies of each family [14], [15]. BlastN searches [42] were performed, with default parameters, using as query each one of the consensus-canonical sequences of each LTRr family, providing a list of coordinates of putative LTRr copies in the genome. Next, different LTRr copies were assigned to the same family following these rules: (1) they present at least a contiguous stretch of 400 bp of the pol and/or gag region with an identity of at least 90% with the family consensus-canonical sequence; or (2) they present an identity of at least 90% over at least half of the LTR sequence length.

### *In silico* mapping of Ty3/gypsy insertions

Chromosomal locations of all the Ty3/gypsy copies in the AgamP3 assembly were obtained through the genome browser of VectorBase [19]. The recent availability of the approximate coordinates delimiting the different chromatin regions in the AgamP3 assembly [23] allowed us to determine the euchromatic type. Briefly, boundaries of PH were identified in the polytene chromosome 2 (bands 19E-20A), chromosome 3 (37D-38A), and in the X (band 6); DIH in regions of 2L (band 21A) and 3L (band 38C); and CIH in a region of 2.9 Mb of 3R (band 35B). Additionally, we defined the PE as the 3 Mb region of the euchromatin of each chromosome arm proximal to the PH, which involves euchromatic regions in 2L (coordinates 2,487,770–5,042,389 and 6,015,228–6,460,609), 2R (55,969,802–58,969,802), 3L (1,896,830–4,235,209 and 5,133,257–5,794,878), 3R (49,131,026–52,131,026), and X (16,928,574–19,928,574). Accordingly, we defined NPE as the euchromatic region of each chromosome excluding the PE. The term “pericentromeric region” refers to the chromosomal region extending from PE to PH.

### Estimation of TEs density along 2L

We used RepeatMasker (http://www.repeatmasker.org/) to search any trace of any known TE family in the genome of *An. gambiae* (PEST), which are those registered in RepBase [41] and TEfam (http://tefam.biochem.vt.edu/tefam/index.php). Then, average density of TEs was estimated as percentage of base pairs for each range of 50 Kb occupied by TEs.

### SV and divergence analyses

The SV and the divergence were estimated after alignments of each copy with the consensus sequence of the corresponding LTRr family. These alignments were performed manually, using the results of the BlastN described above as a guide, with the help of the sequence editor BioEdit version 5.0.9 [43]. In some cases, unreliable alignments were obtained avoiding a correct assessment of their SV and/or divergence. The probable cause of these mismatches is mosaic evolution, which is known to have played an important role in the molecular evolution of Ty3/gypsy group in *An. gambiae* [14] and *Drosophila* [44].

For a description of the SV we first followed the criteria defined by Kaminker et al. [21], which considered as partial any LTRr copy less than 97% of the length of the canonical-consensus sequence of their corresponding family. Nevertheless, an important fraction of the Ty3/gypsy elements analyzed contains tracks of Ns, representing unsequenced gaps that mask the LTRr sequence, making the estimate of their length unapproachable. Therefore, we alternatively redefined the category “partial” as any LTRr copy for which the sum of the length of the deletions exceeds 3% of the length of the respective canonical-consensus sequence. Additionally, we defined two other categories that provide further information about the level of degradation of an element: (1) “moderately-fragmented”, which comprises those LTRr copies showing 1 or 2 indels ≥10 bp with respect to the consensus sequence of the corresponding LTRr family; and (2) “highly-fragmented”, which comprises those presenting ≥3 indels ≥10 bp. As a consequence, those LTRrs without indels ≥10 bp, presenting the same length as the consensus sequence of the corresponding family, were considered complete. Those insertions bearing identity exclusively to the LTR of a family consensus sequence were considered solo-LTRs. Those insertions with unsequenced gaps but meeting the criteria to be regarded as putatively complete based on the analysis of the available sequence were classified as “unknown” condition.

The divergence between each LTRr copy and the corresponding consensus was estimated as the proportion of nucleotide differences with the aid of MEGA version 2.1 [45], using the pairwise deletion option.

### Gene-LTRr association analysis and gene density estimation

We defined a gene-LTRr association as an LTRr located within the transcription borders of a gene or within 1000 bp upstream or downstream of a gene [37]. All the Ty3/gypsy LTRrs located in the NPE were submitted to this analysis. BioMart [46] was employed to identify these associations by comparison of our insertion coordinates and the gene coordinates in the AgamP3 dataset (AgamP3.5 gene set) of Ensembl [47]. Finally, we used the Ensembl genome browser to elucidate if insertions were located in an intron or exon.

Gene density was measured dividing the number of genes contained in each region and the total number of base pairs of the region. Gene content was recruited from both Ensembl and VectorBase [19], [47].

### Study sites and mosquito collections

We studied populations from Kenya and Burkina Faso. In Kenya (East Africa), the study sites include: (1) Asembo Bay (in the text referred to as Asembo), located on the shores of Lake Victoria in Western Kenya; and (2) Jego, located 700 km away, on the coast of the Indian Ocean near the Tanzanian border. The mosquitoes belonging to molecular form S were collected from both sites in 1987 [48]. The DNA samples analyzed in this study include 46 mosquitoes from Asembo and 19 mosquitoes from Jego. In addition, DNA samples from 10 mosquitoes of *An. arabiensis* collected in Asembo were also analyzed. Both populations, Asembo and Jego, are separated by the Great Rift Valley, which represents a restrictive barrier to gene flow within the species *An. gambiae* [49]. In Burkina Faso (West Africa), the study site was Goundry, where mosquitoes were collected in 2001. The DNA samples analyzed include 50 mosquitoes of the species *An. gambiae* form M, 48 of *An. gambiae* form S and 10 mosquitoes of *An. arabiensis*.

### Loci selection for the occupation rate analysis

The occupation rate analysis of a set of selected insertions was carried out in order to assess the possible role of genetic drift in the evolution of divergent Ty3/gypsy insertions in the *An. gambiae* genome [14]. The selection of putative ancient insertions was prioritized as follows: first, those copies 7%–10% divergent relative to the consensus were recruited; among these divergent insertions, and to facilitate the PCR process, those displaying shorter size (i.e., 150–300 bp), and without indels and/or unsequenced gaps, were selected for the PCR optimization process. A total of 13 insertions passed these filters, although only 3 of them overcame the PCR optimization process satisfactorily (Figure S1). In addition, the occupation rate of five low divergent insertions (Table S3) without indels and unsequenced gaps (Tubio et al., unpublished data) was determined for comparisons.

### Primer design, genotyping and sequencing

In general, two different PCRs and, therefore, two different sets of primers were designed per LTRr locus selected for the occupancy rate analysis. The first set of primers, named F1 and R1 (forward1 and reverse1, respectively), were designed outside the TE copy and flanking it. For the second set of primers, named F1 and R2 (forward1 and reverse2, respectively), the reverse primer was designed inside the copy. All primers were designed with the aid of GeneFisher [50], using default parameters. PCR reactions were as follows: 0.6 µM primer forward, 0.6 µM primer reverse, 1.5 mM Mg^+2^, 0.16 mM dNTPs, 1x Taq buffer and 1.25 Units of Taq polymerase were mixed in a total reaction volume of 25 µl. The PCR cycle conditions were as follows: one cycle of 95°C for 5 min, followed by 35 cycles of 95°C for 30 sec, annealing temperature for 30 sec, 72°C for 45 sec; and finally one extension extra cycle of 72°C for 10 min. For the screening analysis, amplicons were visualized in agarose gels stained with ethidium bromide. For those three loci selected for the extensive analysis (*GQ468823*, *GQ468822*, and *GQ468821*) genotyping was carried out in a 3730 Applied Biosytems sequencer, using GeneScan 500 LIZ ladder (Applied Biosystems) for a correct assignment of the amplicon size. The sequences of the primers, annealing temperatures and expected amplicons sizes are detailed in Table S4. For sequencing, the amplicons were extracted from 3% agarose electrophoresis gels and purified using QIAquick Gel Extraction Kit (Qiagen). Sequencing PCR reactions were performed using Bigdye v3.0 (Applied Biosystems). Dyes were removed using an ethanol-based protocol, and PCR products were diluted in deionized formamide. Sequencing was carried out in a 3730 Applied Biosytems sequencer.

## Supporting Information

Dataset S1 **Characterization of the Ty3/gypsy complement of** ***An. gambiae.*** This Excel file contains information relative to the characterization of 1045 insertions of the Ty3/gypsy group in the AgamP3 assembly. Column 1 indicates the Ty3/gypsy lineage and column 2 the name of the Ty3/gypsy family [15]. Column 3 indicates the scaffold where each insertion was identified. The coordinates (first and last positions) of each insertion in a scaffold are shown in columns 4 and 5. Columns 6, 7, 8 and 9 indicate results of the *in silico* mapping: column 6 the chromosome (Chr), columns 7 and 8 the coordinates, and column 9 the cytogenetic division/subdivision. Column 10 shows the classification of each insertion according to two categories: proviral or solo-LTR (“unknown” means that classification was not possible due to the presence of unsequenced gaps). Column 11 indicates the sequence divergence (Div) from the consensus. Finally, column 12 indicates the SV relative to the consensus sequence. Three different types of SV were studied: “del” means “deletion”, “ins” means “insertion”, and “dup” means “duplication”. “No” means “no SV”. “ND” means “not determined”. SV events were annotated as follows: different SV events are separated by a comma; the size of the SV event in base pairs is indicated after each SV type; the symbols “(5′)” and “(3′)” after SV events indicate that the SV affects, respectively, the LTR5′ or LTR3′. If two different SV events occur at the same nucleotide position they are separated by a dash.
(XLS)

Dataset S2 **TE density along chromosome 2L.** This excel file shows the density of total TEs in ranges of 50 Kb along the 2L arm. Columns 1 and 2 indicate the coordinates along the chromosome. Column 3 gives the estimation of TE density.
(XLS)

Table S1 **Average divergence of proviral copies and solo-LTRs.** This table shows the average divergence and standard deviation of the total number of proviral and solo-LTR insertions of each chromosome for which it was possible to determine their divergence relative to the consensus. “N” indicates the number of insertions analyzed (data recruited from Dataset S1).
(PDF)

Table S2 **Ty3/gypsy LTRr-gene associations in** ***An. gambiae.*** This file shows all the associations detected between Ty3/gypsy retrotransposons and genes in the *An. gambiae* genome. Column 1 indicates the chromosome where each association was detected. Columns 2 and 3 give the coordinates of each insertion associated to a gene. Column 4 indicates the classification of each insertion according to two categories: proviral and solo-LTR (“unknown” means that classification was not possible due to the presence of unsequenced gaps). Column 5 gives the sequence divergence from the consensus. Column 6, 7 and 8 indicate the VectorBase gene identifier (AGAP number) of the associated gene and the coordinates in the genome. Column 9 shows the proximity to a gene in base pairs (proximity of 0 bp means that the insertion is located within a gene). Column 10 indicates the region of the gene affected: “exon” or “intron” if the insertion is located within transcription boundaries, and “5′” and “3′” if the insertion is located, respectively, downstream or upstream of a gene. Finally, Column 11 shows the putative function of the gene involved in the association, if known, according to Ensembl [47].
(XLS)

Table S3 **Occupancy rate of Ty3/gypsy LTRrs in** ***An. gambiae.*** We successfully analyzed the frequency insertion profiles of eight Ty3/gypsy LTRr loci in natural populations of *An. gambiae*. Column 1 shows the name assigned to each locus analyzed (loci 6, 7 and 8 also show Gene Bank accession numbers). Column 2 indicates the lineage and family the selected loci belong to. Column 3 gives the LTRr location by chromosomal arm, coordinates, and cytogenetic division and subdivision (number and letter in parenthesis, respectively). Column 4, the chromatin type (NPE, non-pericentromeric euchromatin; IH, intercalary heterochromatin; n. d., not determined). Column 5, the structural condition (proviral or solo-LTR). Column 6 indicates the divergence of each insertion from consensus. Last columns display information relative to the results of the occupancy rate analysis. Column 7 the type of analysis performed. Columns 8 and 9 show the occupancy rate from 0.0 to 1.0 and, in parentheses, the frequency.
(PDF)

Table S4 **Primer sequences and PCR conditions.** This table reveals the conditions for the PCR amplification of the eight Ty3/gypsy-loci selected for the occupation rate analysis. Column 1 shows the name assigned to each locus analyzed (loci 6, 7 and 8 also show Gene Bank accession numbers). Columns 2 displays information relative to the primers employed in locus amplification (see Methods). Primers F1 of loci 6, 7 and 8 were labelled with FAM in the 5′ extreme. Column 3 shows the expected amplicon size and column 4 the annealing temperatures (T).
(PDF)

Figure S1 **Selection of Ty3/gypsy ancient insertions for the occupation rate analysis.** This graphic shows the distribution, according to their size (Y axis) and divergence relative to consensus (X axis), of those Ty3/gypsy loci lower than 500 base pairs and without indels and unsequenced gaps. Only those insertions presenting a size of 150-300 bp and without indels of, at least, 10 bp and/or without unsequenced gaps were finally selected for PCR validation. Red dots represent loci not selected for the PCR optimization process. Yellow dots represent loci selected for the PCR optimization process, but finally discarded due to amplification difficulties. Green dots represent those putative loci selected for the occupation rate analysis (Table S3).
(TIF)
