# Supplementary material for: Evolutionary Dynamics of the Ty3/Gypsy LTR Retrotransposons in the Genome of Anopheles gambiae
Source: PLoS One. 2011 Jan 24;6(1):e16328. doi: 10.1371/journal.pone.0016328 (PMC3026039; doi:10.1371/journal.pone.0016328)
Supplement: Table S1 — Average divergence of proviral copies and solo-LTRs. This table shows the average divergence and standard deviation of the total number of proviral and solo-LTR insertions of each chromosome for which it was possible to determine their divergence relative to the consensus. “N” indicates the number of insertions analyzed (data recruited from Dataset S1). (PDF) [file pone.0016328.s003.pdf]

**Arm**		**Solo-LTR**	**Proviral**
3R	Average	0.034 ± 0.031	0.009 ± 0.015
	N	43	57
3L	Average	0.034 ± 0.030	0.016 ± 0.020
	N	36	69
2R	Average	0.032 ± 0.031	0.017 ± 0.023
	N	41	102
2L	Average	0.038 ± 0.030	0.017 ± 0.024
	N	35	93
X	Average	0.032 ± 0.026	0.017 ± 0.027
	N	36	63
Total	Average	0.034 ± 0.030	0.016 ± 0.022
	N	191	384
