# Supplementary material for: Evolutionary Dynamics of the Ty3/Gypsy LTR Retrotransposons in the Genome of Anopheles gambiae
Source: PLoS One. 2011 Jan 24;6(1):e16328. doi: 10.1371/journal.pone.0016328 (PMC3026039; doi:10.1371/journal.pone.0016328)
Supplement: Table S3 — Occupancy rate of Ty3/gypsy LTRrs in An. gambiae. We successfully analyzed the frequency insertion profiles of eight Ty3/gypsy LTRr loci in natural populations of An. gambiae. Column 1 shows the name assigned to each locus analyzed (loci 6, 7 and 8 also show Gene Bank accession numbers). Column 2 indicates the lineage and family the selected loci belong to. Column 3 gives the LTRr location by chromosomal arm, coordinates, and cytogenetic division and subdivision (number and letter in parenthesis, respectively). Column 4, the chromatin type (NPE, non-pericentromeric euchromatin; IH, intercalary heterochromatin; n. d., not determined). Column 5, the structural condition (proviral or solo-LTR). Column 6 indicates the divergence of each insertion from consensus. Last columns display information relative to the results of the occupancy rate analysis. Column 7 the type of analysis performed. Columns 8 and 9 show the occupancy rate from 0.0 to 1.0 and, in parentheses, the frequency. (PDF) [file pone.0016328.s005.pdf]

Locus	Lineage (Family)	LTRr location	Chromatin type	Condition	% Div	Analysis	WEST AFRICA		EAST AFRICA	
							M	S	Asembo	Jego
Locus 1	CsRn1 (G52_AG)	2R: 4043596-4047772 (8A)	NPE	Proviral	0.0	Screening	0.0 (0/5)	0.0 (0/5)	0.0 (0/5)	0.0 (0/5)
Locus 2	CsRn1 (G50_AG)	2R: 18580773-18584954 (11A)	NPE	Proviral	0.0	Screening	0.0 (0/5)	0.0 (0/5)	0.0 (0/5)	0.0 (0/5)
Locus 3	CsRn1 (G50_AG)	2R: 27194388-27194533 (12E)	NPE	Solo-LTR	0.0	Screening	0.0 (0/5)	0.0 (0/5)	0.0 (0/5)	0.0 (0/5)
Locus 4	CsRn1 (G51_AG)	2R: 30309166-30315068 (13D)	NPE	Proviral	0.6	Screening	0.0 (0/5)	0.0 (0/5)	0.0 (0/5)	0.0 (0/5)
Locus 5	CsRn1 (G50_AG)	3L: 4901607-4901752 (38C)	IH	Proviral	5.7	Screening	1.0 (5/5)	1.0 (5/5)	1.0 (5/5)	1.0 (5/5)
Locus 6 GQ468823	Mag (G57_AG)	2L: 19195392-19195689 (22F)	NPE	Solo-LTR	7.3	Screening<br>Extensive	1.0 (5/5)<br>1.0 (50/50)	1.0 (5/5)<br>1.0 (48/48)	1.0 (5/5)<br>1.0 (46/46)	1.0 (5/5)<br>1.0 (19/19)
Locus 7 GQ468822	Mdg3 (8967)	UNK	n. d.	Solo-LTR	7.9	Screening<br>Extensive	1.0 (5/5)<br>1.0 (50/50)	1.0 (5/5)<br>1.0 (48/48)	1.0 (5/5)<br>1.0 (46/46)	1.0 (5/5)<br>1.0 (19/19)
Locus 8 GQ468821	Mdg3 (G31_AG)	3R: 43554406-43554585 (35C)	NPE	Solo-LTR	8.2	Screening<br>Extensive	1.0 (5/5)<br>1.0 (50/50)	1.0 (5/5)<br>1.0 (48/48)	1.0 (5/5)<br>1.0 (46/46)	1.0 (5/5)<br>1.0 (19/19)
