# Supplementary material for: Evolutionary Dynamics of the Ty3/Gypsy LTR Retrotransposons in the Genome of Anopheles gambiae
Source: PLoS One. 2011 Jan 24;6(1):e16328. doi: 10.1371/journal.pone.0016328 (PMC3026039; doi:10.1371/journal.pone.0016328)
Supplement: Table S4 — Primer sequences and PCR conditions. This table reveals the conditions for the PCR amplification of the eight Ty3/gypsy-loci selected for the occupation rate analysis. Column 1 shows the name assigned to each locus analyzed (loci 6, 7 and 8 also show Gene Bank accession numbers). Columns 2 displays information relative to the primers employed in locus amplification (see Methods). Primers F1 of loci 6, 7 and 8 were labelled with FAM in the 5′ extreme. Column 3 shows the expected amplicon size and column 4 the annealing temperatures (T). (PDF) [file pone.0016328.s006.pdf]

Locus	Primer (5´ to 3´)		Amplicon	T
Locus 1	F1	CCC CTC GAG GAT AAG ACA	743	54
	R2	CAT TCA ATC CGG CAG CA		
Locus 2	F1	CAG TGA CGT CCC TAG CA	749	53
	R2	TTG TTC CGT GAG CTC GA		
	F1	CAG TGA CGT CCC TAG CA	711	56
	R1	GCG ATA CCG TGC GTC A		
Locus 3	F1	ATG CTT CCG AAG AGA GGA	604 / 750	55
	R1	TCC GTA CTG CAA GAG GTA		
Locus 4	F1	TTG GCC CTC TGG CGT A	676	56
	R2	TCG ATG ATG GGG GGG AA		
Locus 5	F1	TAT GTT CAC TGC GGC AA	256 / 402	55
	R1	TGG AGC TGA ACG TCT GA		
Locus 6 (GQ468823)	F1	^{FAM}TCA CAA CAG CCG AAC GA	600	60
	R1	ATG GAC TGC CGC CCT A		
	F1	^{FAM}TCA CAA CAG CCG AAC GA	185	59.1
	R2	TCC CGA TCG GCA CTC A		
Locus 7 (GQ468822)	F1	^{FAM}CGT TTT GCC CAT GCC TA	577	56.9
	R1	TTG ACC ATG CGG CAG A		
	F1	^{FAM}CGT TTT GCC CAT GCC TA	246	60
	R2	CGG AAC GGA AAA GAC GA		
Locus 8 (GQ468821)	F1	^{FAM}TGG AAT CAC ACT CCA CGA	491	60
	R1	CGG AAG TTG TGC TAG CAA		
	F1	^{FAM}TGG AAT CAC ACT CCA CGA	275	60
	R2	CGT TCT GTT CGC TGT CA		
